# Induction of Labor in Protracted Pregnancy

**Published:** 1880-10

**Authors:** P. W. Van Peyma


### INDUCTION OF LABOR IN PROTRACTED PREGNANCY.

BY P. W. VAN PEYMA, M. D

THE object of this short paper is twofold: First, to insist upon and emphasize the fact that cases of protracted pregnancy do occur; and secondly, to show that some of these cases, being recognized as such, are properly treated by the induction of labor.

That pregnancy is occasionally protracted beyond the recognized limit of 278 to 280 days, we believe to be quite generally admitted. Still, this asumption may not be allowed to pass unchallenged, and we will therefore fortify our position by quoting at some length a number of well-known writers upon obstetrics:

Cazeaux devotes a chapter to the subject of retarded labor, in the course of which he says: "As an ordinary rule the pregnancy terminates about the two hundred and seventieth day after conception. However, labor often occurs at an earlier period than this, and on the other hand, it may not appear until some time in the course of the tenth month, or even at the termination of this period, although the latter is a much more unusual circumstance.

After speaking of Mr. Tessier's observations upon cows and mares, submitted to the Academy of Science, at Paris, he adds: "the question would still remain undetermined, if careful observations directly made, and well made, on the human species had not removed all doubt on that point. For several cases bearing on this subject now enrich our science, where a single well-established instance would suffice to produce conviction." Cazeaux then proceeds to quote as an example, Desormaux's case, in which pregnancy was protracted to nine calendar months and a fortnight.

Bedford also quotes this case and adds: "I cite this case to show that nature does sometimes exceed the ordinary period of 280 days, or 40 weeks," and he adds: "there are numerous other cases recorded by authors of equal probity, exhibiting not only the occasional protraction of gestation, &c.;" but he further adds, "that Dr. Simpson records, as having occurred in his own practice, cases in which the period reached 336, 332, 324 and 319 days; Dr. Merriman, 278 days, and Prof. Murphy, 297 days. Dratles reports two cases which nearly equaled 356 days each, and Prof. Meigs publishes a case which he deems entirely trustworthy of 420 days. Dr. Bedford's conclusion is that the precise duration of pregnancy is not positive but simply relative.

Leishman relates a case in which delivery occurred 295 days after a single coitus. The weight of the child was 12 lbs. and 3 oz.

Dr. Playfair, after speaking of the Gardner peerage case, says: "Since that time many apparently perfectly reliable cases have been recorded in which the duration of gestation was obviously much beyond the average, and in which all sources of fallacy were carefully excluded." He calls attention to Simpson's cases and adds, "Indeed the experience of most accoucheurs will parallel such cases which may be more common than is generally supposed, inasmuch as they are only likely to attract attention when the husband has been separated from the wife beyond the average and expected duration of the pregnancy." Playfair also cites some cases in which pains coming on at the expected time subsided, and the labor did not occur until a month after, or at over 300 days from the supposed commencement of pregnancy.

Taylor in his medical jurisprudence, and other writers, give similar testimony. It is admitted that the cessation of menstruation and quickening are not always reliable data, but the existing evidence is sufficient that liberal allowance can be made for occasional errors. The testimony brought forward is believed to be sufficient for our purpose, and is considered proof that pregnancy is occasionally protracted beyond the usual period of about 40 weeks. We now come to the second proposition, viz.: cases of protracted pregnancy, sometimes occurring and admitting of recognition as such, are sometimes properly treated by the induction of labor at the usual time of parturition, if not before. As an example I submit the following case.

Mrs. H., aged 27, pregnant for the third time. First labor normal. During the course of the second pregnancy she became convinced that gestation was being protracted beyond the usual period. The physician in attendance, one of recognized standing, while at first skeptical, appears to have become satisfied of the fact after about two weeks' delay, and accordingly induced labor by means of internal remedies, presumably ergot. Within twenty-four hours labor was excited, but was difficult and completed only after long delay and the use of forceps. The child weighed over 12 lbs.

July 10th I was called to see Mrs. H. in her third pregnancy, and informed of the previous history as given. Upon making an examination I could not satisfy myself that gestation was completed. The patient informed me that she ceased to menstruate on the 19th of September, and also affirmed that she had never suffered from irregular menstruation. Although 294 days had here elapsed, I still advised delay, and to this the patient reluctantly submitted. On the 25th of July I was again called and found Mrs. H. very nervous, nearly hysterical over her condition. She implored me to do something for her. After mature deliberation I decided to induce labor. My objects were two: first, to relieve the patient's mind, she being quite sick with apprehension and worry; and, secondly, to deliver her of a smaller child. The os uteri being dilated sufficiently to admit a finger, introduced a flexible bougie, No. 9, between the uterine walls and the membranes, a distance of about six or seven inches. The remainder of the instrument was curled up in the vagina. Pains were soon excited, and the labor progressed slowly but normally until the os was dilated to the size of an orange, when it being quite rigid and the pains weak and inefficient, the forceps were applied and the labor completed with their aid. The child was quite large and fully developed; weight not determined. The date of birth 27th of July, which shows three hundred and eleven days to have elapsed since the cessation of menstruation.

The question is, was the procedure justifiable? As the sequel proves no harm was done. On the other hand were not some good results obtained? The child was quite certainly smaller than it would have been at any subsequent time. The mother was spared the anxiety of anticipating a severe labor, the result of an overgrown child.

While I admit that this procedure is very liable to abuse, I also claim that, as in this case, it has its advantages. Certainly the induction of labor in certain cases is considered justifiable—for example, in deformed pelvis, placenta praevia, excessive vomiting, &c. Why not in these cases? Why less when the head promises to be too large, than when the pelvis is too small? Playfair says: "I shall only briefly refer to a few more uncommon causes which occasionally necessitate its performance. One of these is a habitually large, or over-firmly ossified fœtal head. Should we meet a case in which the labors are always extremely difficult, and the head apparently of unusual size, although there is no apparent want of space in the pelvis, the induction of labor would be perfectly justifiable, and in all probability would accomplish the desired object. In such cases the full period of delivery would require to be anticipated by a very short time. A week or a fortnight might make all the difference between a labor of extreme severity and one of comparative ease. If it is justifiable to anticipate the full term, it must certainly be so to interfere when this time is reached or passed.

The data obtained from the cessation of menstruation, and from quickening being admitted as occasionally misleading, the trouble will be found in determining exactly when the full term is reached. This being determined, the course of action seems clearly to be the induction of labor at the proper time.

